# Gastrointestinal stromal tumor of the stomach with lymph node metastasis treated by laparoscopic and endoscopic cooperative surgery with lymph node pick-up resection: A case report and literature review

**DOI:** 10.1016/j.ijscr.2020.10.131

**Published:** 2020-11-02

**Authors:** Akira Kubota, Shirou Kuwabara, Kenzi Yamaguchi, Kazuaki Kobayashi, Hideki Hashidate

**Affiliations:** a Department of Digestive Surgery, Niigata City General Hospital, 463-7 Shumoku, Chuo-ku, Niigata City, Niigata 950-1197, Japan; b Department of Pathology, Niigata City General Hospital, 463-7 Shumoku, Chuo-ku, Niigata City, Niigata 950-1197, Japan

**Keywords:** Gastrointestinal stromal tumor, Laparoscopy and endoscopic cooperative surgery, Lymph node metastasis, Case report

## Abstract

**Introduction:**

Gastrointestinal stromal tumor of the stomach with lymph node metastasis is a rare condition, and few cases have been treated by gastrectomy and systematic lymph node dissection.

**Presentation of case:**

We report the case of a patient who was treated by local resection with laparoscopic and endoscopic cooperative surgery and lymph node pick-up resection. A 73-year-old woman presented with gastric submucosal tumor and swollen lymph node. The submucosal tumor was 30 mm in size. After surgery, the tumor was confirmed to be a gastrointestinal stromal tumor by immunostaining. No recurrence was noted 36 months following the surgery.

**Discussion:**

Laparoscopic and endoscopic cooperative surgery allows minimal resection because the exact resection margin can be identified. Therefore, endoscopy is a useful aid in the treatment of gastric gastrointestinal stromal tumor. The MIB-1 index of metastatic lymph node was lower than that of a primary lesion; hence, the prognosis might not depend on lymph node metastasis.

**Conclusion:**

Laparoscopic and endoscopic cooperative surgery and lymph node pick-up resection were useful and minimally invasive. Systematic lymph node dissection is not a viable option.

## Highlights

• We performed LECS with pick-up lymph node dissection for GIST of the stomach with lymph node metastasis.
• LECS with pick-up lymph node dissection allows minimal gastric resection for GIST of the stomach with lymph node metastasis.
• The endoscopy was useful for giving us exact resection margins using this magnification view.

## 1 Introduction

Gastrointestinal stromal tumor (GIST) of the stomach with lymph node metastasis is a rare disease [1]. In such cases, the primary lesion is usually large. Therefore, gastrectomy and systematic lymphadenectomy may be performed [[2], [3], [4], [5], [6], [7], [8], [9]]. However, it has not been conclusively shown that gastrectomy and systematic lymphadenectomy are necessary. We have performed partial gastrectomy with laparoscopic and endoscopic cooperative surgery (LECS) and lymph node pick-up resection for a GIST of the stomach with lymph node metastasis. In this report, we present the case and review relevant literature.

## 2 Presentation of case

This case report is presented in accordance with the SCARE 2018 guidelines [10]. Its unique identification number in the Research Registry can be provided upon request.

**Fig. 1:**
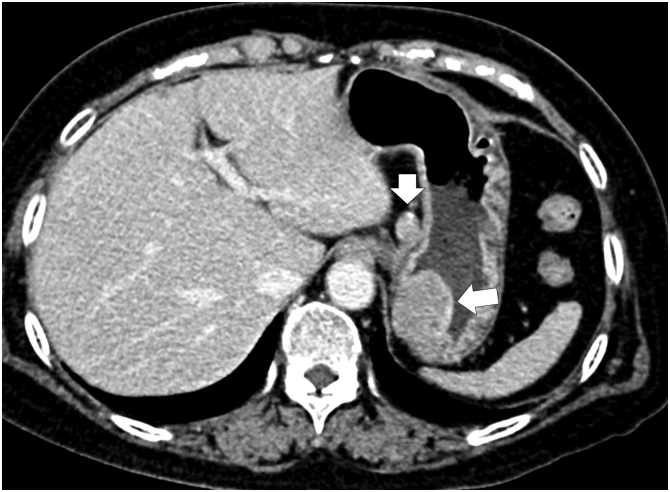
Abdominal computed tomography. A submucosal tumor 30 mm in diameter is connected to the gastric wall, and a 14 mm solid mass shows contrast effect in the early phase.

**Fig. 2:**
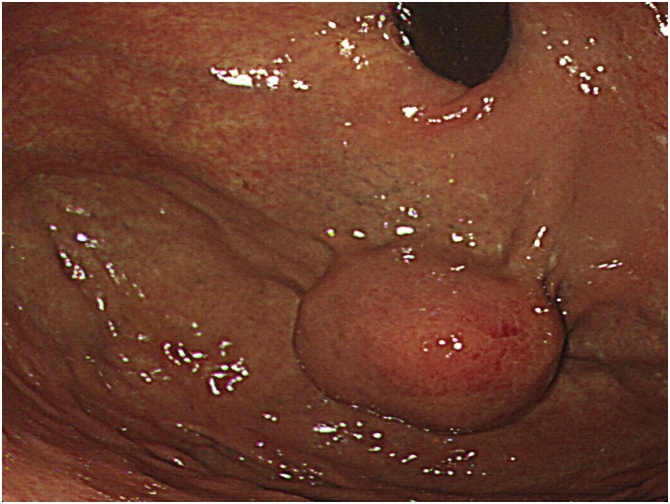
Esophagogastroduodenoscopy findings. A submucosal tumor of the stomach, 30 mm in diameter in the upper anterior wall of the stomach with no ulcerations.

**Fig. 3:**
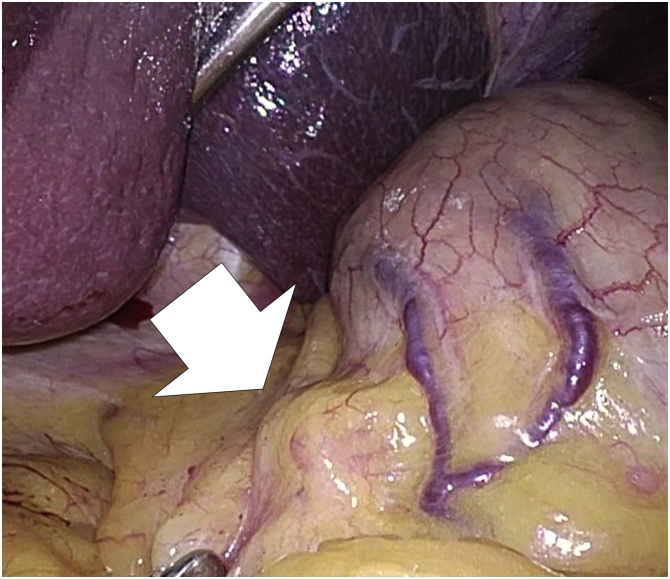
Laparoscopic findings. Swollen lymph node near the primary tumor.

A 72-year-old female patient with respiratory distress was referred to our hospital. A gastric submucosal tumor (SMT) measuring 30 mm and swollen lymph node were found by computed tomography (CT) during investigation for respiratory distress (Fig. 1). SMT was located on the upper anterior wall side of the stomach, and the swollen lymph node was in the lesser omentum near the primary tumor. Past medical history revealed pulmonary thromboembolism and no abdominal surgeries. Blood tests revealed no abnormalities or increases in tumor markers. No relevant drug use, family history data or psychosocial factors were identified. Esophagogastroduodenoscopy revealed a 30 mm SMT in the upper anterior wall side of the stomach. Ulceration was not observed (Fig. 2). Preoperative diagnosis was gastric SMT with lymph node metastasis. We performed LECS and lymph node pick-up resection according to the current international medical guidelines (Fig. 3) [11,12].

Histopathological examination showed a gastric SMT, which was 33 × 32 × 25 mm. Hematoxylin and eosin staining showed a solid nodular mass with no evidence of necrosis. Immunohistochemically, c-kit, CD34, and DOG1 were positive, while SMA and S-100 proteins were negative. Venous invasion was seen; however, there was no lymphatic invasion. Based on these findings, the primary tumor was diagnosed as GIST. The MIB-1 index was 5%, and modified Fletcher classification indicated intermediate risk [13]. The size of the lymph node was 14 × 10 × 14 mm. It was a solid, mesenchymal tumor with calcification and lymphatic tissue. Immunohistologically, c-kit, CD34, and DOG1 were positive, similar to gastric tumors. Thus, it was diagnosed as lymph node metastasis of GIST. The MIB-1 index of the lymph node was 2%–3%.

The patient was discharged from the hospital on postoperative day 5 without complications. Imatinib was not administered as postoperative adjuvant therapy; however, no recurrence was noted at 36 months post-surgery. Written informed consent was obtained from the patient for publication of this case report. This case report was approved by the ethics committee of Niigata City General Hospital.

## 3 Discussion

We reviewed the published literature since 2000 on GIST of the stomach with lymph node metastasis to reveal the clinical features and examine the validity of LECS and lymph node pick-up resection. We performed literature searches with Google scholar and PubMed from 2000 to 2018. The keywords used were “GIST,” “stomach,” and “lymph node metastasis.” Eight studies [[2], [3], [4], [5], [6], [7], [8], [9]] and 15 case reports were found (Table 1).

**Table 1:** Summary of cases of GIST of the stomach with lymph node metastasis in the literature.

Citation (years)	Age	Sex	Surgical procedure	Tumor diameter	Fission image	Number of lymph node metastasis	Lymph node dissection	Imatinib	Distant metastasis	Recurrence	Prognosis
Kubo (2017) [7].	68	M	TG	5.0	20/50 HPF	1	D1	Yes	Yes	Liver	23 m dead
Claudia (2011) [4].	13	F	Subtotal	7.0	4/50 HPF	Multiple	D1	Yes	Liver	Liver	24 m alive
Tokunaga (2011) [8].	32	M	PG	9.0	Unknown	1	D1	Unknown	Yes	Unknown	12 m dead
	46	F	TG	9.0	Unknown	6	D2	Unknown	No	Unknown	33 m dead
	55	M	PG	5.5	Unknown	1	D2	Unknown	No	Unknown	39 m dead
	35	M	PG	6.0	Unknown	1	D2	Unknown	No	Unknown	47 m dead
	68	M	TG	13.0	Unknown	1	D1	Unknown	Yes	Unknown	11 m dead
Valadao (2008) [5].	54	F	Wedge resection	27.0	2/50 HPF	1	D1	Yes	No	Liver	Alive
	42	M	TG	9.0	25/50 HPF	1	D1	Yes	No	Liver	Alive
	32	F	Subtotal	8.5	18/50 HPF	5	D1	Yes	No	Liver	Alive
Canda (2008) [2].	32	F	DG	8.0	25/50 HPF	7	D1	Yes	No	No	12 m alive
Sato (2007) [6].	78	M	PG	4.0	15/50 HPF	1	D1	Unknown	No	Unknown	Unknown
	40	F	Wedge resection	3.2	3/50 HPF	3	Pick-up	Yes	Liver	Unknown	Unknown
Asakage (2007) [9].	63	F	TG	8.0	26/50 HPF	7	Unknown	Yes	No	Liver	3 m alive
Catani (2007) [3].	43	M	TG	Unknown	Unknown	Unknown	Unknown	Yes	Liver, lung	Liver	12 m alive
Our case (2018)	63	F	Wedge resection	3.3	10/50 HPF	1	Pick-up	No	No	No	24 m alive

TG: total gastrectomy, PG: proximal gastrectomy, DG: distal gastrectomy, HPF: high power field.

We performed a partial gastrectomy with LECS and lymph node pick-up resection. GISTs of the stomach with lymph node metastasis are very rare [1]. Previous studies reported surgical treatment with gastrectomy and systematic lymphadenectomy [[2], [3], [4], [5], [6], [7], [8], [9]]. According to a recent review of current international clinical guidelines by JNCCS and ESMO [11,12], the recommended surgical strategy is as follows. First, if the margin can be preserved, partial gastrectomy including endoscopic surgery is suggested. Second, prophylactic lymph node dissection is not necessary; pick-up dissection of lymph nodes suspected of metastasis is deemed sufficient [11,12]. Moreover, according to a recent study, prophylactic lymphadenectomy is associated with poor survival in patients with GIST [14].

The prognostic factor might be not lymph node metastasis but tumor size and the MIB-1 index, as described in several risk classifications (Modified Fletcher, Fletcher, and Miettinen) [11,13]. GIST of the stomach with lymph node metastasis is reported to often present a tumor larger than 5 cm [1]. The tumor size in 12 patients (80.0%) was not less than 5 cm; among the 12 patients, 6 (50%) died. Additionally, the tumor size in two other patients was less than 5 cm. There were no deaths in these cases. In the present case, the MIB-1 index of metastatic lymph nodes was not higher than that of the primary tumor. The MIB-1 index of metastatic lymph nodes was described in only one case [7]. The result of that case was consistent with ours and exhibited a satisfactory prognosis.

In this case, imatinib was not administered, but no recurrence was seen 36 months post-surgery. The reason was as follows: the risk was classified as intermediate as per the Modified Fletcher classification [11,13], R0 resection was performed, and the MIB-1 index of the metastatic lymph node was lower than that of the primary tumor. However, more cases are required to determine if administration of imatinib is warranted in such cases, including pathological considerations of metastatic lymph nodes.

In the present case, endoscopy was helpful for identifying the exact resection margin. According to Hiki et al., LECS was safe with a shorter operative time, less bleeding and adequate resection margin [15]. The patient had no stress on food intake, due to minimal resection.

## 4 Conclusion

Partial gastrectomy with LECS and pick-up resection of lymph nodes might be useful and efficient. Furthermore, LECS allows minimal gastric resection because the exact resection margins can be identified under the endoscopic magnification view.

## Funding

The authors have no sources of funding to report.

## Ethical approval

This study has been approved by our hospital’s ethical board, following its clinical ethics regulations. The approval reference number is 20-002.

## Consent

We hereby state that we have obtained both verbal and written consent from the patient. This consent was given both for the procedures performed and for the present publication of the results of said procedures. Records of this consent are in the power of our hospital’s administration.

## Author contribution

Dr. Shirou Kuwabara revised the manuscript. Dr. Kenzi Yamaguchi performed the operation. Dr. Hideki Hashidate did the pathological diagnosis. All authors read and approved the final manuscript.

## Registration of research studies

1. Name of the registry: Not applicable.
2. Unique identifying number or registration ID: Not applicable.
3. Hyperlink to your specific registration (must be publicly accessible and will be checked): Not applicable.

## Guarantor

I, (Akira Kubota) hereby take full responsibility for the present study. I have access to the original data and patient’s information, was involved in the development of the study and had a determinant part in the decision to publish.

## Provenance and peer review

Not commissioned, externally peer-reviewed.

## Declaration of Competing Interest

The authors declare that they have no competing interests.

## References

[1] De Matteo R.P., Lewis J.J., Leung D., Mudan S.S., Woodruff J.M., Brennan M.F. (2000). Two hundred gastrointestinal stromal tumors recurrence patterns and prognostic factor for survival. Ann. Surg..

[2] Canda A.E., Ozsoy Y., Nalbant O.A., Sagol O. (2008). Gastrointestinal stromal tumor of the stomach with lymph node metastasis. World Surg. Oncol..

[3] Catani M., De Milito R., Simi M. (2005). New orientations in the management of advanced metastatic gastrointestinal stromal tumors (GIST): combination of surgery and systemic therapy with imatinib in a case of primary gastric location. Chir. Ital..

[4] Otto C., Agaimy A., Braun A., Rädecke J., Hoeppner J., Illerhaus G., Werner M., Kontny U., Halleret F. (2011). Multifocal gastric gastrointestinal stromal tumors (GISTs) with lymph node metastases in children and young adults: a comparative clinical and histomorphological study of three cases including a new case of Carney triad. Diagn. Pathol..

[5] Valadão M., de Mello E.L., Lourenço L., Vilhena B., Romano S., Castro Ldos S. (2008). What is the prognostic significance of metastatic lymph nodes in GIST. Hepatogastroenterology.

[6] Sato T., Kanda T., Nishikura K., Hirota S., Hashimoto K., Nahagawa S., Ohashi M., Hatakeyama K. (2007). Two cases of gastrointestinal stromal tumor of the stomach with lymph node metastasis. Hepatogastroenterology.

[7] Kubo N., Takeuchi N. (2017). Gastrointestinal stromal tumor of the stomach with axillary lymph node metastasis. World J. Gastroenterol..

[8] Tokunaga M., Ohyama S., Hiki N., Fukunaga T., Yamamoto N., Yamaguchi T. (2011). Incidence and prognostic value of lymph node metastasis on c-Kit-positive gastrointestinal stromal tumor of the stomach. Hepatogastroenterology.

[9] Asakage N., Kobayashi S., Gotou T., Sasaki M., Tsukada K., Suzuki T. (2007). Two cases of gastrointestinal stromal tumor (GIST) of the stomach and a consideration of its malignancy potential and treatment strategy-report of two cases. Gan To Kagaku Ryoho.

[10] Agha R.A., Borrelli M.R., Farwana R., Koshy K., Fowler A., Orgill D.P., For the SCARE group (2018). The SCARE 2018 statement: updating consensus surgical CAse REport (SCARE) guidelines. Int. J. Surg..

[11] Demetri G.D., Benjamin R.S., Blanke C.D., Blay J.Y., Casali P., Choi H. (2007). NCCN Task Force report: management of patients with gastrointestinal stromal tumor (GIST)-update of the NCCN clinical practice guidelines. J. Compr. Canc. Netw..

[12] Casali P.G., Abecassis N., Aro H.T., Bauer S., Biagini R., Bielack S. (2018). ESMO Guidelines Committee and EURACAN. Gastrointestinal stromal tumours: ESMO-EURACAN Clinical Practice Guidelines for diagnosis, treatment and follow-up. Ann. Oncol..

[13] Nishida T., Blay J.Y., Hirota S., Kitagawa Y., Kang Y.K. (2016). The standard diagnosis, treatment, and follow-up of gastrointestinal stromal tumors based on guidelines. Gastric Cancer.

[14] Li C., Su D., Xie C., Chen Q., Zhou J., Wu X. (2019). Lymphadenectomy is associated with poor survival in patients with gastrointestinal stromal tumors. Ann. Transl. Med..

[15] Hiki N., Yamamoto Y., Fukunaga T., Yamaguchi T., Nunobe S., Tokunaga M., Miki A., Ohyama S., Seto Y. (2008). Laparoscopic and endoscopic cooperative surgery for gastrointestinal stromal tumor dissection. Surg. Endosc..

